# Seropositivity for Influenza A(H1N1)pdm09 Virus among Frontline Health Care Personnel

**DOI:** 10.3201/eid1901.111640

**Published:** 2013-01

**Authors:** Kumar Alagappan, Robert A. Silverman, Kathy Hancock, Mary Frances Ward, Meredith Akerman, Fatimah S. Dawood, Alicia Branch, Sandra De Cicco, Evelene Steward-Clark, Megan McCullough, Karen Tenner, Jacqueline M. Katz

**Affiliations:** North Shore–Long Island Jewish Health System, New Hyde Park, New York, USA (K. Alagappan, R.A. Silverman, M.F. Ward, S. De Cicco, M. McCullough, K. Tenner);; Hofstra North Shore–Long Island Jewish School of Medicine, Hempstead, New York, USA (K. Alagappan, R.A. Silverman, M.F. Ward);; Albert Einstein College of Medicine, Bronx, New York, USA (K. Alagappan, R.A. Silverman);; Feinstein Institute for Medical Research, Manhasset, New York, USA (K. Alagappan, R.A. Silverman, M.F Ward, M. Akerman);; and Centers for Disease Control and Prevention, Atlanta, Georgia, USA (K. Hancock, F.S. Dawood, A. Branch, E. Steward-Clark, J.M. Katz)

**Keywords:** influenza A(H1N1)pdm09 virus, influenza A(H1N1)pdm09, influenza, viruses, seropositivity, emergency department service, hospital, occupational exposure, pandemic, health care personnel

## Abstract

Seroprevalence of antibodies to influenza A(H1N1)pdm09 virus among 193 emergency department health care personnel was similar among 147 non–health care personnel (odds ratio 1.4, 95% CI 0.8–2.4). Working in an acute care setting did not substantially increase risk for virus infection above risk conferred by community-based exposures.

Transmission of infectious disease from acutely ill patients to health care personnel (HCP) is a critical concern during disease outbreaks. During the initial months after the emergence of influenza A(H1N1)pdm09 virus, comparisons to prior pandemic viruses (*1*) and reports of increased illness and death in younger adults (*2*,*3*) heightened concerns about the safety of frontline HCP caring for patients with A(H1N1)pdm09 and the ability of the health care system to meet demands for health care services if infected HCP had to stay home from work.

New York, New York, was one of the first densely populated areas in the United States to experience outbreaks of A(H1N1)pdm09. These early outbreaks and the concomitant surge in patient volumes in our emergency department (ED) provided the opportunity to evaluate and compare risk for A(H1N1)pdm09 virus infection among frontline HCP and non-HCP from the same community in a virus-naive population before availability of the A(H1N1)pdm09 monovalent vaccine.

## The Study

Written informed consent was obtained and the study approved by the Human Subjects Review Board of the Feinstein Institute for Medical Research of the North Shore–Long Island Jewish Health System. Long Island Jewish Medical Center and the adjoining Cohens Children’s Hospital are tertiary care teaching hospitals in Queens, New York. During April 24–June 11, 2009, the volume of all-cause ED visits to these 2 institutions increased by 62% compared with the same period during 2008. There were 5,100 visits with influenza-like illness (ILI) as the primary manifestation, which coincided with a surge of ILI visits to EDs throughout New York, New York (*4*).

HCP who worked in an acute care or specially designated influenza area during April 24–June 11, 2009, were asked to participate in our study during October 28–December 16, 2009, by completing a survey and submitting a blood sample. During the same time, we enrolled a convenience sample of non-HCP adults ≥18 years of age residing in the same region as HCP. None of the participants received the A(H1N1)pdm09 monovalent vaccine before enrollment. Assuming a 20% seroprevalence of antibodies to A(H1N1)pdm09 among the general population and a type I error probability of 5% and type II error probability of 20% (power 80%), a sample size of 140 HCP and 140 non-HCP would be sufficient to show a 15% difference in seroprevalence between HCP and non-HCP.

Serum samples were tested by using hemagglutination inhibition and microneutralization assays with A/Mexico/4108/2009, an A/California/07/2009 (H1N1)–like virus (*5*). Participants with a single serum sample with a microneutralization titer ≥40 and a hemagglutination inhibition titer ≥20 were considered seropositive for antibodies to A(H1N1)pdm09 virus. This combination of antibody titers in single convalescent-phase serum samples was shown to provide 90% sensitivity and 96% specificity for detection of A(H1N1)pdm09 infection in persons <60 years of age and 92% specificity in persons 60–79 years of age (*5*).

Separate analyses comparing seropositive and seronegative persons were performed for HCP and non-HCP by using either a χ^2^ statistic, Fisher exact test, or Mann-Whitney test. In multivariable logistic regression models, factors associated with seropositivity in univariate analysis (p<0.10) or hypothesized to be exposure risk factors were included. Analyses were performed by using SAS version 9.2 software (SAS Institute Inc., Cary, NC, USA).

We enrolled 193 HCP and 147 non-HCP in the study. Non-HCP were older (median 47 years, range 18–80 years) than HCP (median 40 years, range 21–65 years) and less likely to recall symptoms of an ILI (Table 1). A similar proportion of HCP and non-HCP reported contact with a household member who had confirmed or suspected A(H1N1)pdm09 and living with children <18 years of age.

**Table 1:** Baseline characteristics of 340 health care personnel tested for seropositivity to influenza A(H1N1)pdm09 virus*

Characteristic	No. (%) health care personnel, n = 193	No. (%) non–health care personnel	p value
Sex			
M	70 (36.3)	68 (46.3)	0.07
F	123 (63.7)	79 (53.7)	NA
Age, y			
<30	35 (18.1)	21 (14.3)	0.01
30–40	58 (30.1)	27 (18.4)	NA
41–50	48 (24.9)	33 (22.5)	NA
51–60	43 (22.3)	49 (33.3)	NA
>60	9 (4.7)	17 (11.6)	NA
Age, y (dichotomized)			
<60	184 (95.3)	130 (88.4)	0.02
>60	9 (4.7)	17 (11.6)	NA
Children <18 y of age in home	85 (44.0)	55 (37.4)	0.22
Received seasonal influenza vaccine during 2008 or 2009	190 (98.5)	123 (83.7)	<0.01
Contact with household member with confirmed or suspected A(H1N1)pdm09	39 (20.2)	23 (15.7)	0.26
Clinical contact with confirmed or suspected A(H1N1)pdm09	146 (75.7)	NA	NA
Health care worker role			
Attending physician	47 (24.4)	NA	NA
Resident physician	30 (15.5)	NA	NA
Registered nurse	58 (30.1)	NA	NA
Other†	58 (30.1)	NA	NA
Cared for primarily adults	126 (65.3)	NA	NA
Cared for primarily children	67 (34.7)	NA	NA
Influenza-like symptoms‡ during spring–summer 2009	81 (42.0)	53 (36.1)	0.27
ILI§ during spring–summer 2009	27 (14.0)	7 (4.8)	0.01
ARI¶ during spring–summer 2009	48 (24.9)	30 (20.4)	0.33

*NA, not applicable; ILI, influenza-like illness; ARI, acute respiratory illness. †Patient care technicians, registrars, and other emergency department staff. ‡Any of the following: fever, cough, body aches, chills, headache, fatigue, runny nose, sore throat, diarrhea, nausea, and vomiting. §Fever AND either cough or sore throat. ¶Two or more of the following: fever, cough, runny nose, sore throat.

Among 193 HCP, 41 (21.2%) were seropositive for antibodies to A(H1N1)pdm09 virus; of these, 12 (29.3%) reported no influenza-like symptoms during the study period. Age, sex, and HCP role were not associated with seropositivity. However, a higher proportion of attending physicians who took care of children were seropositive than those who took care of adults (30.8% vs. 8.7%; p<0.07). Seropositive HCP did not work more ED shifts than seronegative HCP (mean 20 vs. 22 shifts; p = 1.0) or temporary influenza treatment center shifts (mean 8 vs. 5 shifts; p = 0.5) during April 24–June 11, 2009. The proportion of seropositive HCP who reported contact with a patient with suspected or confirmed A(H1N1)pdm09 was similar (76.3% vs. 73.2%; p = 0.9).

Among 147 non-HCP, 24 (16.3%) were seropositive for antibodies to A(H1N1)pdm09 virus. A higher proportion of persons living with children <18 years of age were seropositive (54.2% vs. 34.2%; p = 0.06) than those not living with children <18 years of age. However, this finding did not reach statistical significance.

Among the 340 study participants, 65 (19%) were seropositive for antibodies to A(H1N1)pdm09 virus. HCP were no more likely to be seropositive than were non-HCP (21.2% vs. 16.3%; p = 0.30). In a multivariate model that included age, sex, receipt of seasonal influenza vaccine, having children <18 years of age in the household, and occupation, only living in a household with children <18 years of age was associated with being seropositive (Table 2).

**Table 2:** Univariate and multivariate analysis of risk indicators for seropositivity for influenza A(H1N1)pdm09 virus among 340 study participants*

Characteristic	No. (%) seropositive, n= 65	No. (%) seronegative, n = 257	Crude OR (95% CI)	Adjusted OR (95% CI)
Sex				
M	22 (33.9)	116 (42.2)	Referent	NA
F	43 (66.2)	159 (57.8)	1.41 (0.80–2.49)	NA
Age, y				
<30	15 (23.1)	41 (14.9)	2.01 (0.60–6.81)	NA
30–40	20 (30.8)	65 (23.6)	1.57 (0.48–5.15)	NA
41–50	14 (21.5)	67 (24.4)	1.15 (0.34–3.86)	NA
51–60	12 (18.5)	79 (28.7)	0.84 (0.25–2.85)	NA
>60	4 (6.2)	22 (8.0)	Referent	NA
Age, y (dichotomized)				
<60	61 (93.9)	253 (92.0)	1.32 (0.44–3.96)	NA
>60	4 (6.2)	22 (8.0)	Referent	NA
Received seasonal influenza vaccine during 2008 or 2009	61 (93.9)	252 (91.6)	1.30 (0.43–3.91)	NA
Contact with person with suspected A(H1N1)pdm09 virus infection	13 (20.0)	49 (17.8)	1.17 (0.59–2.31)	NA
Children <18 y of age at home	36 (55.4)	104 (37.8)	1.96 (1.13–3.40)	1.96 (1.13–3.40)
Occupation				
Non-HCP	24 (36.9)	123 (44.7)	Referent	NA
HCP	41 (63.1)	152 (55.3)	1.35 (0.77–2.36)	NA
ILI† during spring–summer 2009	15 (23.1)	19 (6.9)	3.97 (1.85–8.49)	NA
ARI‡ during spring–summer 2009	25 (38.5)	53 (19.3)	2.55 (1.41–4.60)	NA

*OR, odds ratio; NA, not applicable; HCP, health care personnel; ILI, influenza-like illness; ARI, acute respiratory illness. ILI and ARI were not included in the adjusted logistic regression model because they did not reflect risk for exposure. †Fever AND either cough or sore throat. ‡Two or more of the following: fever, cough, runny nose, sore throat.

## Conclusions

We found that 21% of frontline HCP working in the ED or specially designated influenza areas during the first wave of A(H1N1)pdm09 virus circulation in New York, New York, were seropositive for antibodies to A(H1N1)pdm09 virus, similar to non-HCP. Overall, our estimated seroprevalence among HCP and non-HCP was 19%, which was similar to estimates after the first wave of A(H1N1)pdm09 virus circulation from other studies (*6*,*7*). Living with children <18 years of age was the only identified risk indicator for seropositivity.

Among HCP, the reported seroprevalence of antibodies to A(H1N1)pdm09 virus in other countries ranges from 7% to 30% (*8*–*12*). Consistent with findings from our study, seroprevalence among HCP in most studies comparing HCP and non-HCP was similar to that for non-HCP (*10*–*12*). However, some studies identified differences in seroprevalence associated with HCP role and between first-line and second-line HCP (*8*,*11*). Although our study did not have adequate power to detect such differences, we observed a trend toward higher seropositivity among physician providers caring for children.

Several studies documented reduction in spread of influenza by facemask use and handwashing (*13*,*14*). Measures taken to limit the spread of infection during A(H1N1)pdm09 in our hospitals included isolation of patients with ILI upon hospital arrival; HCP use of N95 protective masks, gloves, and gowns; and standard precautions such as handwashing. We did not evaluate the effect of these precautions on risk for A(H1N1)pdm09 virus infection because we did not measure HCP adherence to prevention measures. However, seropositivity for A(H1N1)pdm09 virus might have been higher among HCP in our study if these preventions measures were not in place.

Our study had several limitations. Our control group may not have been representative of the general community. We did not assess for use of influenza antiviral medications after potential exposures among participants. On the basis of our sample size, we only had adequate power to detect a ≥15% difference in seropositivity between HCP and non-HCP. We did not have prepandemic serum samples from study participants to evaluate for pre-existing cross-reactive antibodies to A(H1N1)pdm09 virus. However, we found that that our criteria for seropositivity were highly specific for detection of A(H1N1)pdm09 virus infection.
